# Gut microenvironmental changes as a potential trigger in Parkinson’s disease through the gut–brain axis

**DOI:** 10.1186/s12929-022-00839-6

**Published:** 2022-07-27

**Authors:** Szu-Ju Chen, Chin-Hsien Lin

**Affiliations:** 1 Department of Neurology, National Taiwan University Hospital, College of Medicine, National Taiwan University, Taipei, 100 Taiwan; 2 Department of Neurology, National Taiwan University Hospital Bei-Hu Branch, Taipei, Taiwan; 3 Graduate Institute of Clinical Medicine, College of Medicine, National Taiwan University, Taipei, Taiwan; 4 Institute of Molecular Medicine, College of Medicine, National Taiwan University, Taipei, Taiwan

**Keywords:** Parkinson’s disease, Gut microenvironment, Gastrointestinal inflammation, Impaired intestinal barrier, Gut microbiota, Gut–brain axis

## Abstract

Parkinson’s disease (PD) is the second most common neurodegenerative disease attributed to the synergistic effects of genetic risk and environmental stimuli. Although PD is characterized by motor dysfunction resulting from intraneuronal alpha-synuclein accumulations, termed Lewy bodies, and dopaminergic neuronal degeneration in the substantia nigra, multiple systems are involved in the disease process, resulting in heterogenous clinical presentation and progression. Genetic predisposition to PD regarding aberrant immune responses, abnormal protein aggregation, autophagolysosomal impairment, and mitochondrial dysfunction leads to vulnerable neurons that are sensitive to environmental triggers and, together, result in neuronal degeneration. Neuropathology studies have shown that, at least in some patients, Lewy bodies start from the enteric nervous system and then spread to the central dopaminergic neurons through the gut–brain axis, suggesting the contribution of an altered gut microenvironment in the pathogenesis of PD. A plethora of evidence has revealed different gut microbiomes and gut metabolites in patients with PD compared to unaffected controls. Chronic gut inflammation and impaired intestinal barrier integrity have been observed in human PD patients and mouse models of PD. These observations led to the hypothesis that an altered gut microenvironment is a potential trigger of the PD process in a genetically susceptible host. In this review, we will discuss the complex interplay between genetic factors and gut microenvironmental changes contributing to PD pathogenesis.

## Background

Parkinson’s disease (PD) is the second most common neurodegenerative disorder, affecting 1–3% of the population over 60 years of age worldwide, with increasing prevalence attributed to the global increase in life expectancy [1, 2]. The classical scenario of PD is characterized by progressive motor symptoms, including rest tremor, bradykinesia, rigidity, and postural instability [3]. Nevertheless, recent research has shown that PD is a heterogenous disorder involving multiple system degeneration and leading to various motor and non-motor manifestations that compromise quality of life, such as sleep disorder, neuropsychiatric symptoms, autonomic malfunction, and sensory symptoms [4]. The pathological hallmark of PD is intraneuronal accumulations of misfolded alpha-synuclein, termed Lewy bodies, along with neuroinflammation, leading to dopaminergic neuronal degeneration in the substantia nigra [3]. Intriguingly, in some patients, Lewy body pathology was first detected outside the brain, starting from neurons in Auerbach’s and Meissner’s plexuses of the gut enteric nervous system (ENS), as well as the olfactory bulbs [5]. This distribution of alpha-synuclein pathology could explain early non-motor symptoms of PD, especially constipation, which can precede motor symptoms by decades [6]. Based on these pathological findings, Braak and colleagues suggested active retrograde transport of alpha-synuclein from the ENS to the brainstem via the dorsal motor nucleus of the vagus nerve [7]. This hypothesis is supported by data from rodent and non-human primate experimental models [8] in which different forms of misfolded alpha-synuclein were transported via the vagus nerve, reaching the dorsal motor nucleus of the vagus nerve in the brainstem, after injection into the intestinal wall [9–11]. Moreover, misfolded alpha-synuclein has been proposed to be capable of self-propagation in a prion-like manner, transferring the pathology to unaffected cells through cell-to-cell transmission through different routes, including endoplasmic reticulum-associated vesicle secretion, exosomes, tunneling nanotubes, and passive diffusion, by promoting the misfolding and spread of alpha-synuclein [12]. However, the molecular and cellular processes that trigger the misfolding and fibrillization, Lewy body formation, and spread of alpha-synuclein from the ENS into the brain remain poorly understood.

The complex interplay between aging and genetic and environmental risk factors collaboratively promotes the development of PD [3]. Over the past few decades, the advent of genetic technology has identified more than 20 PD-causative genes in familial forms of PD [13]. The identification of these genes shed light on the molecular mechanisms causing alpha-synuclein aggregation and neuronal degeneration, including mitochondrial dysfunction, increased oxidative stress, autophagolysosomal impairment, and aberrant intracellular vesical trafficking and neuroinflammation [14]. Among these PD-causative genes, mutations in the gene encoding leucine-rich repeat kinase 2 (*LRRK2*) is the most prevalent for both familial and sporadic forms of PD. However, fewer than 50% of the mutation carriers will end up developing the disease by 80 years of age [15], indicating that factors other than the genetic mutation are needed to trigger the PD process. The gastrointestinal tract is one of the routes by which environmental substances enter the body, and the enteric neurons residing in proximity are protected only by a thin epithelial cell layer [7]. Given the neuropathological evidence that Lewy bodies may start in the ENS [5], it is reasonable to raise the hypothesis that an altered gut microenvironment is a potential trigger of the PD process in genetically susceptible hosts.

In this review, we summarize recent evidence of altered gut microenvironments in the PD process. We also discuss the complex interplay between genetic predisposition and potential gut microenvironmental triggers in the pathogenesis of PD. As the gastrointestinal tract is involved in the early disease stage, elucidating the pathophysiological process that takes place in the very beginning gut prodromal stage of PD will not only unveil the disease mechanism, but provide future directions in the development of novel diagnostic biomarkers and disease-modifying therapy that could mitigate disease progression.

## Clinical evidence of gastrointestinal system involvement in PD

Although the hallmark clinical symptom of PD is motor dysfunction (i.e., rigidity, bradykinesia, resting tremor) when there is at least 60% neuronal loss in the substantia nigra (SN), clinical features of patients with PD also present with versatile non-motor symptoms, such as REM sleep behavior disorder, depression, idiopathic anosmia, and constipation [4]. These non-motor PD symptoms can occur years before the onset of classical motor symptoms [16]. Thus, they are now considered prodromal clinical markers of early disease stages according to the International Movement Disorders Society [17]. Gastrointestinal dysfunction has been well-described, and constipation is the most prevalent and earliest pre-motor symptom in the prodromal phase of the disease [18]. Prospective studies have consistently revealed that populations with constipation have a long-term increased risk of PD, by up to one to threefold over 10 years [6, 19–24]. In a systemic analysis, the odds ratio for developing PD was estimated to be 2 to 2.5 in a group of participants with constipation compared to those without constipation [6]. Notably, the prevalence and severity of constipation worsens as disease progresses, with one study reporting up to 82% of advanced-stage patients having constipation [24]. Although the frequency of constipation varies greatly between studies depending on the diagnostic criteria applied and study design, it estimated to be 27 to 66% in PD patients and 5 to 34% in the general population [6, 24]. These clinical observations are in line with the neuropathological findings that Lewy body pathology is first detected in the neurons of the ENS in the gut [5]. In addition to the clinical symptom of constipation, recent studies have identified that inflammatory markers are elevated in both the colonic tissues and feces of patients with PD compared to controls, indicating evidence of intestinal inflammation in PD [25–28]. Epidemiology studies have revealed an increased risk of PD in patients with inflammatory bowel disease (IBD) [29, 30] and reduced risk of developing PD if they receive anti-inflammatory treatment [31]. These observations suggest that, in addition to a vagal nerve connection, the gut could also participate in the PD process through the inflammatory response [25]. These pieces of epidemiological evidence have led to the hypothesis that gastrointestinal dysfunction contributes to PD. The close connection of the gastrointestinal tract with the environment offers a gateway between environmental triggers and the brain [32].

## Histopathological evidence of gastrointestinal involvement in PD

**Fig. 1:**
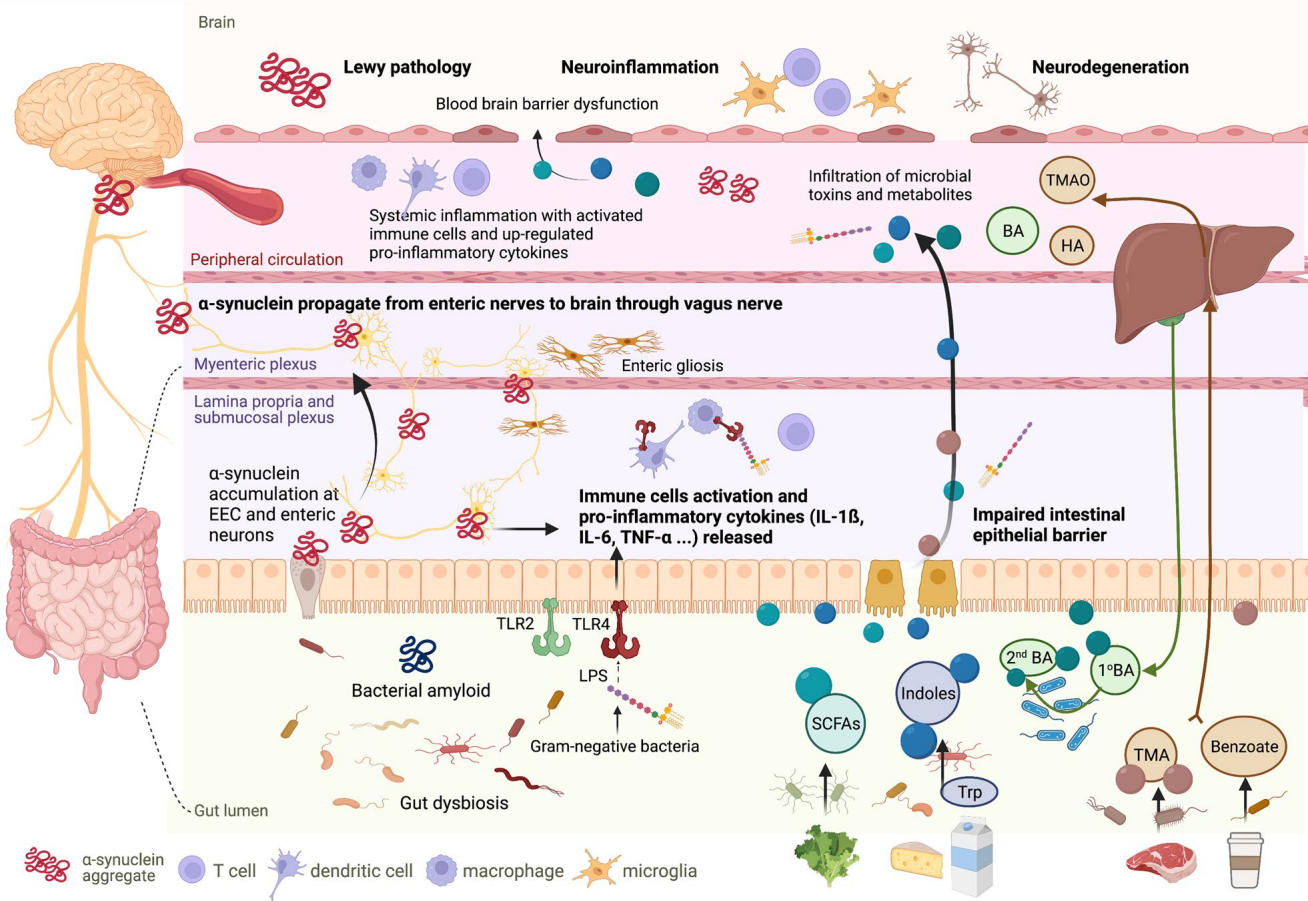
Schematic of gut microenvironmental changes in PD and their contribution to disease pathophysiology. Enteric α-synuclein deposition, gastrointestinal inflammation, and intestinal epithelial barrier dysfunction are observed in PD. Pathological α-synuclein is found in enteroendocrine cells (EECs) and enteric neurons, which propagates through the vagus nerve to the brainstem, resulting in Lewy pathology in the central nervous system. Enteric α-synuclein also induces inflammatory responses involving activation of the caspase-1-dependent inflammasome and production of pro-inflammatory cytokines, leading to intestinal barrier malfunction [87]. Meanwhile, alteration of the gut microbiota composition in PD may accelerate α-synuclein aggregation, partly through secretion of bacterial amyloid [101, 117, 197]. Gut dysbiosis may also trigger enteric inflammation and increased intestinal permeability with systemic infiltration of microbial toxins and metabolites that activate and modulate immune responses and promote PD pathogenesis [85, 103]. Intestinal and systemic inflammation involving increased expression of TLR-4, T cells, and associated pro-inflammatory cytokines (e.g., TNF, IL-1ß, IL-6) results in greater permeability of the blood–brain barrier, neuroinflammation, and dopaminergic neuronal degeneration [64, 74, 166, 198]. BA, bile acid; HA, hippuric acid; IL-1ß, interleukin-1ß; TNF, tumor necrosis factor; TLR, toll-like receptor; TMA, trimethylamine; TMAO, trimethylamine N-oxide; Trp, tryptophan; LPS, lipopolysaccharide; SCFAs, short chain fatty acids

The gastrointestinal tract is one of the largest interfaces between the host and environment in humans. The gut is also referred to as the second brain in the human body. Large numbers of neurons are contained in the gut ENS, a count that is similar to the number of neurons in the spinal cord, with 20 neuronal and 4–7 glial cell types [33]. This gut neural network extends beyond enteric neurons and is comprised of enteric glia, neurons of peripheral ganglia innervating the gut, intrinsic neurons, and specialized innervated epithelial sensors, such as enteroendocrine cells, which constitute the “gut connectome” [34]. The gut neurons are divided into two systems. One is an intrinsic ENS comprising the myenteric and submucosal plexuses, and the other is extrinsic innervation that emanates from the central nervous system (CNS) and communicates with the gut via sympathetic and parasympathetic inputs [34]. The interaction between the gut and brain is a bi-directional communication between the ENS and CNS [35]. The gut–brain axis is composed of neuroendocrine and neuroimmune systems, including the hypothalamic–pituitary–adrenal axis, sympathetic and parasympathetic arms of the autonomic nervous system (including the ENS and vagus nerve), and the residents within the gut lumen, the gut microbiota [35]. Several pathological findings could provide explicit evidence that the gastrointestinal tract and ENS are involved in the disease process of PD, including aggregation of misfolded alpha-synuclein within the ENS, intestinal inflammation, and impaired gut barrier integrity. These manifestations develop in the early stage of the disease and are attributed to the pathogenesis of PD (Fig. 1).

### Alpha-synucleinopathy in the gastrointestinal tract

Post-mortem pathology studies have reported widespread Lewy body pathology in enteric neurons at the intestinal mucosa, submucosa, and myenteric plexus interconnecting the vagus nerve and dorsal motor nucleus of the vagus nerve in the brainstem in both prodromal and symptomatic PD patients [7, 36]. These findings led to Braak’s hypothesis that Lewy body pathology in PD may initiate in the gastrointestinal tract and then propagate to the CNS through the vagus nerve in some patients [7, 36]. Subsequent histopathological studies demonstrated the deposition of pathological alpha-synuclein in the intestinal tissues of both the upper and lower gastrointestinal tract of prodromal or pre-clinical PD patients up to 20 years before the onset of motor symptoms [37–39]. In addition, the dorsal motor nucleus of the vagus nerve was not involved in widespread Lewy body pathology in only 7 to 8.3% of PD patients. In contrast, more than half of the patients with PD had pathological progress adhering to Braak’s theory [40]. Furthermore, inter-neuronal spread of pathological alpha-synuclein has been observed in PD patients with an accumulation of Lewy body in grafted dopaminergic neurons 10 years after receiving fetal dopaminergic neuronal transplantation [41–43]. Evidence from animal and cell models also support the cell-to-cell prion-like transmission of pathological alpha-synuclein that leads to Lewy pathology and dopaminergic neuronal loss [44, 45]. Supporting these findings from human PD patients, data from rodent and non-human primate models have shown that intra-intestinal injection of alpha-synuclein fibrils triggers aggregation of phosphorylated alpha-synuclein at the dorsal motor nucleus of the vagus nerve, followed by the locus coeruleus and substantia nigra via the vagus nerve in a longitudinal follow-up [32, 46, 47]. The pathological alpha-synuclein spread, dopaminergic neuronal degeneration, and motor dysfunction were mitigated by vagotomy [46, 48, 49]. Consistently, epidemiological studies using Swedish and Danish registries have demonstrated that truncal vagotomy decreases the risk of developing PD by 15–22%, further supporting that the vagus nerve is involved in the transmission of pathological alpha-synuclein in the pathogenesis of PD [50, 51].

Notably, Lewy body pathology also spreads by a non-vagal route. Transmission of pathological alpha-synuclein through sympathetic nerves and the peripheral circulation has been observed in rodent and non-human primate models [8, 47, 52]. An increase in plasma alpha-synuclein correlates with disease duration and motor symptom severity in PD patients and to the extent of enteric alpha-synuclein deposition in primate models of PD [8, 53, 54]. In addition, enteric alpha-synuclein is a pivotal modulator of immune responses. Accumulation of intestinal alpha-synuclein occurs most abundantly in the appendix, which is full of lymphatic tissues and responsible for preserving the intestinal immune system in both neurologically normal subjects and patients with PD [55, 56]. Appendectomy was associated with a reduced risk of developing PD in a long-term follow-up but, intriguingly, increased the occurrence of PD shortly after surgery, which may be attributed to intestinal inflammation [57–62]. Studies in cellular and animal models have revealed that alpha-synuclein possesses chemotactic features [63]. Misfolded alpha-synuclein could activate innate immune responses, up-regulate the expression of pro-inflammatory cytokines (e.g., tumor necrosis factor-α (TNFα), interleukin-1ß (IL-1ß)) and promote maturation of antigen-presenting cells, leading to adaptive immune responses [63]. A recent in vivo PD rodent model study further demonstrated that the enteric alpha-synuclein aggregations cause chronic intestinal inflammation and impair gut barrier junction integrity, which may promote neurodegeneration through systemic inflammatory processes [64]. Thus, pathological alpha-synuclein aggregations are observed in the gastrointestinal tract in the early stage of the PD process and may affect the CNS through the vagus nerve and non-vagal immune responses.

### Chronic intestinal inflammation

Intestinal inflammation has been identified as another gastrointestinal feature of PD based on colonic tissue findings and increased inflammatory markers in feces from patients with PD [25–28]. Mild chronic colitis has been detected in the early disease stage [28, 65]. Studies of colon biopsies have shown that the level of expression of inflammatory markers, including toll-like receptor (TLR)-2, TLR4, CD3+ T cells, T helper (Th)1, Th17, and related pro-inflammatory cytokines and chemokines CCL2, CCL5, CCR5, IL-1ß, IL-6, IL-8, IL-17A, interferon (IFN)-ß, IFN-γ, TNF-α associated with glial cell markers is elevated in PD patients compared to controls [28, 66]. Examination of stool immune profiles also found that inflammatory signatures, including calprotectin, IL-1α, IL-1ß, CXCL8, and C-reactive protein, are higher in PD patients than unaffected controls, independent of disease duration, suggesting the participation of intestinal inflammation throughout the disease course [26, 27, 67]. In addition, epidemiological studies have shown that, in both Western and Asian populations, the risk of developing PD is higher in patients with IBD than those without IBD [29, 31, 68–71]. The risk of PD is increased by 28% in patients with Crohn’s disease and 30% in patients with ulcerative colitis, whereas the risk is reduced by 78% in IBD patients who receive anti-TNF-α therapy [30, 31]. Furthermore, recent genome-wide association studies have revealed that PD and IBD share several common pleiotropic genetic loci related to microbial sensing, immunity, and autophagolysosomal function, including *LRRK2* [72, 73].

The mechanism by which chronic colitis promotes PD pathogenesis remains elusive. In vivo studies have demonstrated that intestinal inflammation induces and exacerbates neuroinflammation and dopaminergic neuronal degeneration with or without Lewy pathology in the CNS [74–77]. It is though that TLR4, a pattern recognition receptor, plays a crucial role in mediating the disease process because an elevation of TLR4 in colonic tissues and TLR4-downstream inflammatory mediators [e.g., nuclear factor-κB (NF-κB) and TNF-α] has been observed in PD patients and mouse models of PD. Knockout of TLR4 or treatment with TNF-α antagonist or TLR4 blockers mitigates neuroinflammation, dopaminergic neuronal loss, and motor dysfunction in both cellular and rodent models of PD [66, 76, 78, 79]. As previously mentioned, intestinal inflammation could trigger the expression of enteric alpha-synuclein and vice versa [62, 80], with intestinal inflammation per se increasing the permeability of the blood–brain barrier (BBB), promoting microgliosis, and aggravating neuronal loss through upregulation of systemic pro-inflammatory cytokines and neuroinflammation [74]. Not surprisingly, impaired intestinal barrier and gut dysbiosis are also closely related to enteric inflammation [81, 83]. Notably, enteric gliosis correlating with intestinal inflammation has been observed in human PD patients and animal models of PD [28, 66]. Although the role of enteric glia in the PD disease process is still unclear, they are known to participate in the maintenance of intestinal homeostasis through modulation of gut immunity, intestinal barrier function, and the gut microbiota [83]. Thus, multiple lines of evidence indicate that chronic intestinal inflammation with or without enteric Lewy body pathology promotes neuropathology in the disease process of PD.

### Increased intestinal permeability (leaky gut phenomenon)

The intestinal epithelium forms a defensive physical barrier with a mucus layer to protect the host against environmental toxins and pathogens [81]. The function of the intestinal barrier is regulated by epithelial and immune cells, associated cytokines, and gut microbiota that, when impaired, leads to increased penetration of luminal materials, including bacteria, toxins, and metabolites, into the body, termed “leaky gut syndrome”, which may trigger detrimental systemic responses and endotoxin invasion of the blood circulation [81, 82, 84]. PD patients have reduced expression of tight junctional proteins, including zonula occludens-1 and occludin, in colonic epithelial cells [66, 85, 86]. In a functional study, the concentrations of urine sucralose excreted after oral ingestion of a sugar cocktail were higher in patients with PD than healthy controls, suggesting increased intestinal permeability, possibly due to disrupted intestinal epithelium integrity [66, 85]. Leaky gut syndrome in PD could be an effect of complicated interactions between enteric inflammation, alpha-synucleinopathy, and an altered gut microbiota as increased intestinal permeability and reduced tight junction proteins have been observed to correlate with colonic alpha-synuclein accumulation, fecal inflammatory markers, and gut dysbiosis in PD patients and animal models of PD [66, 85, 87, 88]. Furthermore, disrupted intestinal permeability has been observed early in the disease course before the occurrence of brain pathology in peripheral endotoxin-induced and transgenic models of PD [88, 89]. This leaky gut phenomenon would allow the entry of intestinal luminal contents into the systemic circulation and then across the BBB to promote PD neuropathology in the CNS. Numerous studies have shown higher systemic concentrations of microbial metabolites and elevated endotoxin-related inflammatory markers in PD patients compared to controls, and the levels have been associated with PD severity, supporting the “leaky gut phenomenon” and associated consequences in the PD process [90–92].

## Metagenomic and metabolomic evidence of gastrointestinal involvement in PD

Trillions of bacteria, viruses, protozoa, and fungi residing in the gastrointestinal tract comprise a vast, dynamic ecosystem that interacts with the host [93]. They participate in maintaining homeostasis of the intestinal barrier, immune maturation, nutrition absorption, and metabolism [93]. Recent evidence indicates that the gut microbiota communicates with the brain and is associated with several neurodegenerative disorders, including PD [94]. Alterations in the composition of bacteria, fungi, and viruses and their related metabolites have been described in PD patients, with most studies focusing on bacteria [95–97]. Small intestinal bacterial overgrowth compared to unaffected controls is a common presentation in PD patients, with an estimated prevalence of 46% in a meta-analysis and this associates with worse motor function and complications [98–100]. 16S rRNA gene amplicon surveys to study the bacterial and archaeal community structures and shotgun metagenomic sequencing to carry out non-targeted sequencing of the gut microbiota community are widely used techniques for investigating the associations between changes in the gut microbiota and the development of PD.

### Altered gut microbiota in PD

A plethora of studies have investigated changes in the gut microbiota in patients with PD compared to healthy controls [101–105]. The results of various studies regarding the gut microbiota in PD are heterogenous, possibly due to differences in the study design, genetic background of enrolled participants, geographical region, dietary habits, lifestyle, disease status, co-morbidities, and use of medication [103]. Meta-analyses showed that the relative abundance of anti-inflammatory and short chain fatty acid (SCFA)-producing bacteria at the phylum level, including *Blautia, Coprococcus, Roseburia, Lachnospira, Fusicatenibacter,* and *Faecalibacterium*, are reduced in PD patients compared to controls, whereas, the amount of *Lactobacillus, Bifidobacterium,* and *Akkermansia* is higher in PD patients than unaffected participants from different ethnicities [101–104]. Notably, opportunistic pathogens and pro-inflammatory bacteria at the phylum level, including *Corynebacterium, Porphyromonas, Alistipes, Bacteroides, Escherichia, Megasphaera* and *Desulfovibrio* are also reportedly enriched in PD [103, 106, 107]. In metagenomic research, gene markers from the gut microbiome were found to accurately discriminate PD patients from healthy controls, with most of the identified markers belonging to *Bacteroides* and *Escherichia* species [108]. Although PD medications do affect the structure of the gut microbiota, as levodopa and entacapone correlate with the degree of reduction in SCFA-producing bacteria, these changes have also been observed in drug-naïve early-stage PD patients [96, 106, 109]. Furthermore, the gut microbiota can also influence the metabolism of anti-PD medication levodopa by using an alternative metabolic pathway and potentially reducing the bioavailability of levodopa [110, 111]. A study reported that *Enterococcus faecalis* harbors the gut microbial tyrosine decarboxylase enzyme, which is responsible for the decarboxylation of levodopa into dopamine in the gut [110]. In this regard, the abundance of *Enterococcus faecalis* compromised levodopa uptake and, consistently, the relative abundance of gut microbial tyrosine decarboxylase gene in fecal samples from PD patients was positively correlated with a higher daily levodopa dosage requirement [110]. Another study reported that *Eggerthella lenta* would metabolize dopamine into m-tyramine, thus reducing dopamine bioavailability [111]. These observations suggest that that levels of *Enterococcus faecalis* and *Eggerthella lenta* in the gut may affect the tratment efficacy of levodopa in PD patients.

Changes in the gut microbiota also correlate with disease progression in PD. A decrease in the SCFA-producing microbiota and increase in pro-inflammatory bacteria correlate with motor and cognitive severity in patients with PD [101, 103, 112]. Transplanting the fecal gut microbiota from PD patients leads to worsened motor symptoms in a transgenic rodent model of PD compared to transplantation from healthy donors [113]. A 3-year longitudinal follow-up study of PD patients revealed that a reduced amount of *Roseburia* species predicted faster progression in both motor and non-motor symptoms of PD [114]. SCFA-producing bacteria are considered beneficial to hosts due to their anti-inflammatory effects in the intestine and their role in maintaining gut and BBB homeostasis [115]. Correspondingly, a reduced amount of SCFA-producing bacteria, including *Fusicatenibacter* and *Faecalibacterium*, correlates with elevated fecal inflammatory calprotectin levels in PD patients [116]. Furthermore, enrichment in *Bacteroides* and *Bifidobacterium* has been linked to elevated expression of systemic and fecal inflammatory markers IFN-γ, TNF-α, and neutrophil gelatinase-associated lipocalin in patients with PD [87, 105]. The increased levels of *Lactobacillaceae* and *Bifidobacteriaceae* in PD patients seem paradoxical and require further investigation, as they are usually recognized as probiotics and are used in some clinical trials to treat constipation [117]. In transgenic mouse models of PD, the gut microbiota promotes pathological alpha-synuclein accumulation, neuroinflammation, and dopaminergic neuronal degeneration, along with aggravation of motor and non-motor symptoms, which were alleviated in germ-free conditions [113]. In addition, recent research has shown that *Akkermansia* induces intestinal alpha-synuclein aggregation as a result of mitochondrial calcium overload in intestinal enteroendocrine cells, which were hypothesized to be an initiating site for alpha-synucleinopathy in response to environmental factors because they are connected to enteric nerves and found to have adjacent accumulation of alpha-synuclein proteins [118, 119].

Notably, emerging evidence indicates that protein nucleation and aggregation may be influenced by an extracellular amyloid protein called “curli”, which is secreted by *Escherichia coli*, inducing neuronal deposition of alpha-synuclein in the gut and promoting neurodegeneration [120, 121]. The abundance of *E. coli* at the colonic mucosa correlates with enteric alpha-synuclein deposition in PD patients [85]. Curli is an amyloid protein that bacteria secrete for surface adherence and biofilm formation [122]. Induction of abnormal alpha-synuclein aggregation by curli has been demonstrated in vitro, and ingestion of curli and curli-producing bacteria accelerates enteric and central alpha-synucleinopathy, as well as neuroinflammation, dopaminergic neuronal degeneration, and motor dysfunction, in transgenic rodent models of PD [121, 122]. Therefore, distinct gut microbiota species result in a pro-inflammatory status in the intestinal tract or promote enteric alpha-synuclein aggregation to facilitate the occurrence and progression of PD.

### Altered gut metabolites in PD

Intestinal microorganisms interact with the host through secreted toxins, by-products, and metabolites to modulate immune responses, endocrine secretion, metabolism, and neurotransmission [123]. Analyses of predicted functional pathways of the gut microbiome have shown that metabolism involving lipopolysaccharide (LPS), carbohydrates, amino acids, lipids, energy, cofactors, and vitamins is altered in PD [96, 108, 109, 124–126]. Consistently, gut microbial metabolites have been observed to be different between PD patients and controls in different biofluids or samples, including feces, plasma, and cerebral spinal fluid (CSF) (Table 1). Some gut metabolites could discriminate PD patients from unaffected controls, whereas some correlate with disease severity and progression, suggesting their participation in the disease mechanism and the potential to serve as disease biomarkers.

**Table 1:** Alteration of microbial metabolites in Parkinson’s disease

Study	Participants	H-Y stage^a^	Sample	Metabolite changes in Parkinson’s disease
Short chain fatty acids				
Unger, Germany [132]	34 PD, 34 controls	2.5 (1–4)	Stool	AA↓ PA↓ BA↓ VA5-
Tan, Malaysia [125]	104 PD, 96 controls	2.2 ± 0.5	Stool	BA↓
Aho, Finland [87]	55 PD, 56 controls		Stool	AA- PA↓ BA↓ VA-
Pablo-Fernandez, UK [97]	35 PD, 50 controls		Stool	AA↓ PA↓ BA↓ VA↓
Chen, Taiwan [91]	96 PD, 85 controls	2.3 ± 1.2	Stool	AA↓ PA↓ BA↓ VA-
Yang, China [92]	95 PD, 33 controls	2.2 ± 0.7	Stool	AA↓ PA↓ BA↓ VA-
Ahmed, India [143]	43 Drug-naïve PD, 37 controls	2.0 (1–3)	Plasma	AA↓
Toczylowska, Poland [203]	19 PD, 21 controls	2.6 ± 0.4	Plasma	AA↑
Zhao, China [204]	28 PD, 18 controls		Plasma	VA↓
Shin, South Korea [134]	38 PD, 33 controls	2.0 ± 0.6	Plasma	AA↑ PA- BA-
Wu, China [138]	50 PD, 50 controls	2.5 (0.5)	Plasma	AA- PA↓ BA↓ VA-
Chen, Taiwan [91]	96 PD, 85 controls	2.3 ± 1.2	Plasma	AA- PA↑ BA↑ VA↑
Kim, South Korea [137]	10 PD, 10 controls		Plasma	AA↑
Yang, China [92]	95 PD, 33 controls	2.2 ± 0.7	Plasma	AA↑ PA3↑ BA- VA5-
Yilmaz, USA [154]	20 PD, 20 controls		CSF	AA-
Kumari, India [135]	76 PD, 37 controls		Saliva	AA↑ PA3↑ BA -
Kumari, India [136]	100 PD, 50 controls		Urine	AA- BA↑
Indole derivatives				
Hatano, Japan [145]	35 PD, 15 controls	2.9 ± 1.1	Plasma	IAA↓
Wuolikainen, Sweden [161]	22 PD, 28 controls		Plasma	indole↑
Sankowski, Poland [160]	18 PD, 9 controls	2.9 ± 0.6	Plasma	IS-
Shao, China [152]	223 PD, 237 controls		Plasma	ILA↓
Rosario, German [109]	8 PD, 10 controls		Plasma	IPA↑, IS↓
Chen, Taiwan [146]	56 PD, 43 controls	2.3 ± 1.1	Plasma	Trp- IAA- IPA↑ ILA-
Sankowski, Poland [160]	18 PD, 9 controls	2.9 ± 0.6	CSF	IS-
Luan, China [149]	106 PD, 104 controls		Urine	ILA↑
Cassani, Italy [147]	68 PD, 43 drug naïve PD, 50 controls		Urine	IS↑
Bile acids				
Zhao, China [204]	28 PD, 18 controls		Plasma	GUDCA↓
Yakhine-Diop, Spain [153]	24 PD, 8 controls		Plasma	CA↑, DCA↑, GDCA↑
Shao, China [152]	223 PD, 237 controls		Plasma	CA↑, DCA↑, GDCA↑, TDCA↑, GDCS↑, TCAS↑, GLCAS↑
Chen, Taiwan [146]	56 PD, 43 controls	2.3 ± 1.1	Plasma	CA-, DCA↑, GDCA↑, CDCA-, GCA-, GCDCA-, UDCA-, GUDCA-
Yilmaz, USA [154]	20 PD, 20 controls		CSF	CA-, DCA-, GDCA-, CDCA-, GCA-, GCDCA↑, UDCA-, GUDCA- LCA-, TCA-, TCDCA-, TDCA-, TUDCA-
Li, USA [151]	15 PD, 12 controls		Appendix	DCA↑, LCA↑
Li, USA [151]	15 PD, 12 controls		Ileum	LCA↑
Trimethylamine *N*-oxide related metabolites				
Tan, Malaysia [125]	104 PD, 96 controls	2.2 ± 0.5	Stool	Choline↓ TMA↓ TMAO↓
Ahmed, India [143]	43 Drug-naïve PD, 37 controls	2.0 (1–3)	Plasma	TMA↓
Sankowski, Poland [160]	18 PD, 9 controls	2.9 ± 0.6	Plasma	TMAO↑
Chen, Taiwan [158]	60 PD, 30 controls	3.2 ± 1.8	Plasma	TMAO↑
Chung, South Korea [200]	85 drug-naïve PD, 20 controls		Plasma	TMAO↓
Kim, South Korea [137]	10 PD, 10 controls		Plasma	TMA↓
Sankowski, Poland [160]	18 PD, 9 controls	2.9 ± 0.6	CSF	TMAO-
Kumari, India [135]	76 PD, 37 controls		Saliva	TMA↑ TMAO↑ Choline-
Luan, China [149]	106 PD, 104 controls		Urine	Choline↑, TMAO↑
Hippuric acid related metabolites				
Burte, UK [165]	41 PD, 40 controls		Plasma	Catechol sulfate↓
Okuzumi, Japan [164]	15 *PRKN* PD, 19 controls	2.4 ± 0.7	Plasma	HA↓ catechol sulfate↓
Rosario, German [109]	8 PD, 10 controls		Plasma	HA↑
Chen, Taiwan [146]	56 PD, 43 controls	2.3 ± 1.1	Plasma	HA↑
Trivedi, UK [162]	43 PD, 21 controls		Sebum	HA↑
Wuolikainen, Sweden [161]	22 PD, 28 controls		CSF	Benzoic acid↑

AA, acetic acid; BA, butyric acid; CA, cholic acid; CDCA, chenodeoxycholic acid; CSF, cerebrospinal fluid; DCA: deoxycholic acid; GCA, glycocholic acid; GCDCA, glycochenodeoxycholic acid; GDCA, glycodeoxycholic acid; GDCS, glycodeoxycholic acid 3-sulfate; GLCAS: glycolithocholic acid 3-sulfate; GUDCA, glycoursodeoxycholic acid; HA, hippuric acid; 3-HK, 3-hydroxykynurenine; H-Y stage, Hoehn and Yahr stage; IAA, indole-3-acetic acid; ILA, indolelactic acid; IS, indoxyl sulfate; LCA, lithocholic acid; PA, propionic acid; TCA, taurocholic acid; TCAS, taurocholic acid 3-sulfate; TCDCA, taurochenodeoxycholic acid; TDCA, taurodeoxycholic acid; TMA, trimethylamine; TMAO, trimethylamine *N*-oxide; Trp, tryptophan; TUDCA, tauroursodeoxycholic acid; UDCA, ursodeoxycholic acid; VA, valeric acid.↑, means increased levels; ↓means reduced levels; - means the levels are not changed

^a^Hoehn and Yahr stage was expressed in median (range), median (interquartile range) or mean ± standard deviation

LPS is an endotoxin from the outer membrane of Gram-negative bacteria that activates surface pattern recognition receptors (e.g., TLR4) on epithelial and immune cells with the assistance of LPS-binding protein (LBP) to generate innate immune responses and modulate amyloidogenesis of alpha-synuclein through direct molecular interactions [127–129]. Studies in PD patients have shown that bacteria with endotoxin (e.g., *Escherichia* and *Bacteroides*) are enriched compared to controls, and the increased colonic *E. coli* correlated with reduced plasma levels of LBP along with upregulated enteric alpha-synuclein expression, oxidative stress, and elevated intestinal permeability [85]. Intra-peritoneal injection of LPS triggers intestinal pathology, including increased intestinal permeability and enteric alpha-synuclein accumulation, before the onset of brain pathology in rodent models of PD [88, 130]. In addition, systemic endotoxin injection evokes chronic systemic inflammation, microglia-mediated neuroinflammation, and dopaminergic neuronal degeneration at the substantia nigra [88, 130]. Exposure to endotoxin triggers the production of LBP, and elevated circulating levels of LBP are associated with chronic systemic inflammation [131]. In PD patients, although plasma LBP levels are reduced at an early disease stage, higher plasma LBP concentrations were observed in the later stage to correlate with systemic pro-inflammatory cytokines TNF-α and IL-6, motor symptom severity, and progression [90]. Taken together, these findings indicate the participation of gut dysbiosis-related endotoxin-mediated inflammation in PD pathogenesis and disease progression.

The major SCFAs are butyrate, propionate, and acetate, which are microbial metabolites and bacterial fermentation products of dietary fibers produced in the colon [115]. Being a major energy source of epithelial cells, most intestinal SCFAs are absorbed by colonocytes, leaving only a minority to enter the systemic circulation and pass across the BBB to modulate systemic immune responses and microgliosis [115]. As SCFA-producing bacteria are reduced in PD patients, it is not surprising to find that fecal levels of SCFAs are decreased in PD patients. The fecal levels of SCFAs positively correlate with the abundance of SCFA-producing microorganisms, including *Butyricicoccus* and *Roseburia*, but inversely correlate with *Escherichia*, *Shigella*, *Akkermansia,* and *Bifidobacterium.* Furthermore, reduced fecal levels of SCFAs further correlate with motor and cognitive severity in patients with PD [87, 91, 92, 97, 125, 132]. SCFAs are activators of G protein-coupled receptors and serve as inhibitors of histone deacetylases [115]. They are considered beneficial to the intestinal epithelium because they promote intestinal barrier integrity, mucosal healing, and anti-inflammatory effect [115]. Deficiency in enteric SCFAs is associated with intestinal inflammation, which may be one of the routes by which gut dysbiosis triggers PD pathogenesis [133]. Intriguingly, though SCFA levels in feces are reduced in PD patients, their concentrations in plasma, urine, and saliva were elevated, which may be attributed to increased intestinal permeability, as a higher plasma to fecal SCFA ratio was observed in PD patients and correlated with a pro-inflammatory gut microbiota [91, 92, 134–137]. Associations between increased plasma SCFA concentrations and disease severity were also observed in PD [91, 92, 138]. In addition, oral intake of SCFAs induces microglia activation, neuronal alpha-synuclein accumulation, and dopaminergic neuronal degeneration with motor impairment in transgenic mouse models of PD [113, 139]. Taken together, the evidence shows that excessive SCFAs in the systemic circulation, possibly resulting from the leaky gut phenomenon frequently observed in PD patients, may be detrimental to the disease process through activation of neuroinflammation [91]. Nevertheless, some studies have shown improved dopaminergic neuronal survival in response to SCFA treatment in rodent models [138, 140–143]. As evidence suggests a crucial role of SCFAs in the interconnection between the gut microbiota and neuropathology of PD, further investigations are warranted to explore the molecular mechanisms of SCFAs in the disease process of PD.

Tryptophan is an essential amino acid that is mostly absorbed and catabolized through the kynurenine pathway in the liver, with the remaining 5–10% passing into the colon for degradation by the microbiota into indole derivatives, which are interspecies signaling molecules that modulate gut hormone secretion, intestinal mucosal homeostasis, and systemic inflammation [144]. Studies have shown that plasma levels of indole-3-acetic acid (IAA) are decreased, whereas indole-3-propionic acid (IPA) is elevated, in PD patients [109, 145, 146]. IAA is a ligand of aryl hydrogen receptor (AHR), which regulates innate and adaptive immune responses, including attenuation of pro-inflammatory cytokine expression upon LPS stimulation and T-cell differentiation [144]. In addition to serving as an AHR ligand, IPA also activates pregnane X receptor and enhances intestinal barrier function [144]. In contrast, indoxyl sulfate is the only indole derivative considered toxic with pro-inflammatory features and is decreased in the systemic circulation and elevated in the urine of PD patients [109, 144, 147]. Intriguingly, the concentrations of indole-3-lactic acid have been found to be reduced in the plasma but increased in the urine of PD patients [148, 149]. Although altered microbial catabolism of tryptophan is observed in PD, its exact role in the disease process needs to be further elucidated.

Bile acids (BAs) synthesized in the liver and secreted into the intestine are mostly taken back up in the ileum, whereas those remaining in the gastrointestinal tract are deconjugated and/or dehydroxylated by the gut microbiota to form secondary BAs [150]. Studies have shown that the level of primary BAs is comparable between PD patients and controls. Secondary BAs deoxycholic acid (DCA) and lithocholic acid are increased and correlate with an increase in BA-metabolizing bacteria and reduced expression of BA re-uptake enzymes in the ileum [151]. Higher plasma levels of DCA and its glycine conjugated form (GDCA) are consistently observed in PD patients compared to controls in both Asian and Western populations [146, 152, 153]. Higher GDCA levels in the CSF were also reported in a small cohort of PD patients [154]. In addition to the emulsification of lipid products, BAs are regulators of the gut microbiota, inflammation, and energy metabolism [133]. As conjugated BAs can cross the BBB with the assistance of transporters, deconjugated BAs could enter the CNS by passive diffusion [155]. BAs could also enhance the permeability of the BBB and regulate neurotransmitters [155]. Dysregulated BA levels are generally considered cytotoxic and pro-inflammatory and have been observed in various neurodegenerative diseases, including Alzheimer’s disease and Huntington’s disease, in addition to PD [151, 155, 156].

A shift toward increased proteolysis has been observed in PD; higher concentrations of detrimental amino acid metabolites with pro-inflammatory features, p-cresol and p-cresol sulfate, have been noted in the plasma and CSF of patients with PD. These levels correlate with an increased abundance of pro-inflammatory bacteria and symptoms of constipation [124, 157]. Benzoate-related metabolites (benzoate, hippuric acid, and catechol sulfate) from dietary polyphenol compounds and choline-associated metabolites (choline, phosphocholine, trimethylamine, and trimethylamine-*N*-oxide) in plasma, CSF, urine, and saliva are also different between PD patients and controls. These levels also correlate with disease severity, though some conflicting results have been reported [109, 125, 135, 137, 143, 146, 158–165].

Altered microbial metabolism may reflect diverse interactions between the heterogenous host genetic background and gut environmental factors contributing to disease formation in individual patients. Notably, altered metabolomes are not unique findings in PD, but are also observed in other neurodegenerative diseases, including Alzheimer’s disease and atypical parkinsonism, with some similar and different results indicating common and distinct disease processes of neurodegeneration [152, 166, 167].

## Interplay between genetic predisposition and gut microenvironmental changes in PD

**Fig. 2:**
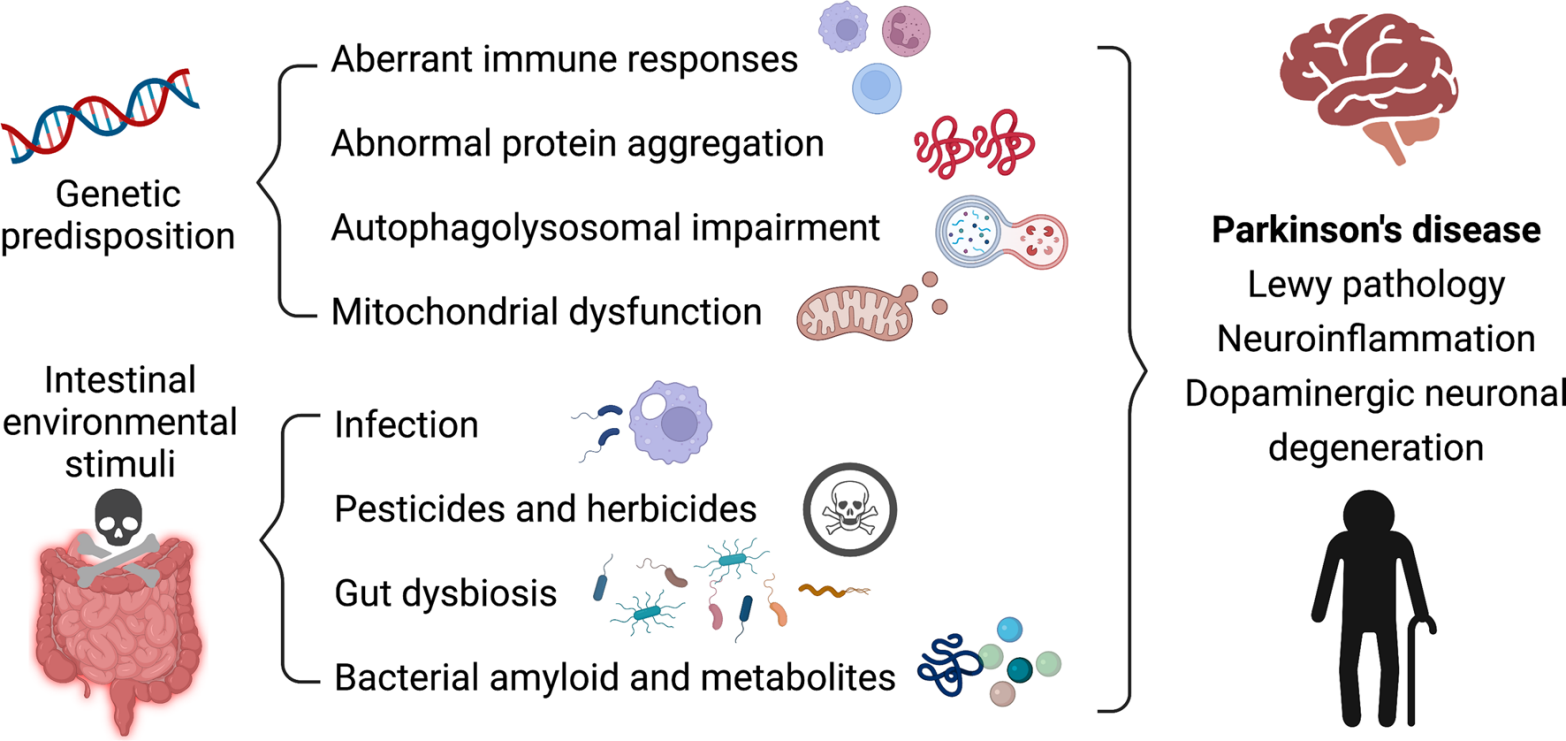
Genetic predisposition and gut microenvironmental changes collaboratively contribute to PD pathogenesis. Genetic susceptibility to PD leads to vulnerable neurons related to aberrant immune responses, abnormal protein aggregation, autophagolysosomal impairment, and mitochondrial dysfunction that together with gut microenvironmental changes attributed to intestinal stimuli (e.g., bacterial infection, exposure to pesticide or herbicide, gut dysbiosis, and altered bacterial amyloid and metabolites) contribute to PD pathogenesis

Genetic mutations contribute to approximately 5 to 15% of patients with PD [13]. Identifying the protein function of these PD-causative genes would lead to a better understanding of the molecular mechanism of dopaminergic neuronal degeneration coming from multiple pathways, including perturbed alpha-synuclein homeostasis, mitochondrial dysfunction, autophagolysosomal impairment, and aberrant immune responses [168]. Though these genetic mutations often cause familial PD, disease penetrance varies widely between mutation carriers, suggesting that non-genetic environmental factors or other genetic modifiers may affect the occurrence of the disease [168]. Notably, the gut microenvironment could be one of the factors that modulates the manifestation of symptoms in genetic forms of PD (Fig. 2). The complex interplay between genetic risk factors in the host and environmental factors, especially gut microorganisms or gut metabolites, has been examined in multiple rodent models of PD (Table 2) [76, 77, 113, 122, 169–173]. In addition, genome-wide association studies have identified numerous genetic risks that increase susceptibility to sporadic PD, and several genes are related to gut microbial regulation and intestinal inflammation, including pattern recognition receptors TLR1 and TLR2, peptidoglycan recognition protein, and MUC2, a component of the mucosal layer that protects the intestinal epithelial barrier [174]. Some of the identified genetic susceptibility to sporadic PD also increases the risk of IBD, among which, *NOD2* is known to be a strong predictor of IBD and to interact with *LRRK2* [175].

**Table 2:** Double-hit rodent models of Parkinson’s disease

PD model	Environmental toxin	Intestinal pathological change		Brain pathological change		Clinical symptoms		Study
		Enteric α-syn^a^	Impaired epithelial barrier	Central α-syn^a^	Neuropathology^b^	GI	Motor	
*SNCA* A53T	Oral paraquat	+		−			−	Naudet [169]
*SNCA* A53T	DSS	+		+	+		+	Kishimoto [77]
*SNCA* A53T	IP LPS				+		−	Vitola [170]
*LRRK2* G2019S, *LRRK2* R1441G	IP LPS				+			Kozina [171]
*LRRK2* G2019S	DSS	+	+	−	+		+	Lin [76]
*PINK1*^±^	IP rotenone				+			Martella [172]
*PINK1*^*−/−*^	Oral bacteria				+		+	Matheoud [173]
Thy1-αSyn	Oral SCFAs			+	+		+	Sampson [113]
Thy1-αSyn	Oral bacteria	+		+	+	+	+	Sampson [122]

DSS, dextran sulfate sodium; GI, gastrointestinal; IP, intraperitoneal; LPS, lipopolysaccharide; PD, Parkinson’s disease; SCFAs, short chain fatty acids; α-syn, α-synuclein. + and − indicates positive and negative results. Blank indicates items not shown

^a^Pathologic α-synuclein deposition at tissue

^b^Neuroinflammation, neuronal loss, and/or synaptopathy at brain

### LRRK2

Mutation of glycine to serine (p.G2019S) in *LRRK2* is the most prevalent PD-causative mutation in both familial and sporadic PD [176, 177]. However, fewer than 50% of *LRRK2* p.G2019S carriers will end up developing the disease at 80 years of age [15], indicating that factors other than the genetic mutation are needed to trigger the PD process. Interestingly, genome-wide association studies have also identified *LRRK2* as a major susceptibility gene for Crohn’s disease, one of the inflammatory bowel disorders [178]. *LRRK2* knock-out mice have prominent colitis compared to wild-type mice upon exposure to chemical stimulants [179]. In addition to high expression in neuronal tissues, *LRRK2* is primarily expressed in immune cells, including macrophages, dendritic cells, and B lymphocytes, in the lamina propria of the gastrointestinal tract [180]. LRRK2 expression is induced by IFN-γ, consistent with the idea that *LRRK2* plays a role in the pathogenesis of gut inflammatory disorder. LRRK2 protein is known to participate in a wide variety of cellular processes, including vesicle trafficking, autophagolysosomal function, and cell proliferation [181]. It can promote the lysozyme-sorting process in Paneth cells and enhance bacterial clearance in *Salmonella* and *Listeria* infection [182, 183]. LRRK2 is also highly expressed in peripheral immune cells and up-regulated during inflammation in response to pro-inflammatory cytokines and activated TLRs [184, 185]. Elevated LRRK2 kinase activity promotes inflammatory responses, including activation of the inflammasome and NF-κB pathway, leading to increased secretion of pro-inflammatory cytokines (e.g., TNF-α and IL-1ß), which assist in pathogen clearance but also worsen colitis, chronic neuroinflammation, and dopaminergic neuronal loss in rodent models [171, 182, 186, 187]. Furthermore, treatment with anti-TNF-α monoclonal antibody greatly reduces gut inflammation and neuroinflammation, mitigates dopaminergic neuronal loss, and alleviates motor symptoms exacerbated by chronic colitis in a transgenic *LRRK2* p.G2019S mouse model [187]. Most of the pathogenic *LRRK2* mutations relating to PD result in hyperactivated kinase activity of LRRK2 protein, and patients carrying the mutation have higher concentrations of pro-inflammatory cytokines in their peripheral circulation [175, 188, 189]. In addition, histopathological studies have demonstrated that LRRK2 expression is up-regulated in colonic biopsy tissue from patients with PD or IBD compared to controls and the expression correlates with disease severity [190–192]. Thus, exaggerated inflammatory responses attributed to pathogenic *LRRK2* mutation upon immune triggers from the gastrointestinal tract contribute to the pathogenesis of PD. Further studies exploring the role of *LRRK2* in the immune system, especially gut innate immunity, are needed to untangle the mechanism of PD beginning from the very early gut prodromal stage.

### SNCA

Missense mutations and multiplication of *SNCA*, the gene encoding alpha-synuclein protein, were among the first identified causes of monogenic PD [168]. Patients carrying pathogenic *SNCA* mutations often have familial PD with early onset and that involves multiple systems with rapid progression [168]. Abundant Lewy body pathology and prominent neuronal loss have also been identified in these patients [168]. Although a detailed mechanism linking pathogenic *SNCA* mutations to PD onset is still being investigated, studies have shown that mutated genes change the structure, aggregation rate, post-translational modification, expression levels, and toxicity of alpha-synuclein, which leads to Lewy body pathology and dopaminergic neuronal loss [193]. In addition to brain pathology, recent studies have found that *SNCA* polymorphisms in PD patients modulate alpha-synuclein aggregation at the ENS before the occurrence of parkinsonian pathological changes in the brain in transgenic mouse models expressing pathogenic *SNCA*, consistent with Braak’s hypothesis [194, 195]. Intriguingly, *SNCA* also regulates the microbiota–gut–brain axis and *SNCA* polymorphisms are associated with alterations in opportunistic pathogens in PD [196]. In support of this finding, intestinal stimulation with oral paraquat, SCFAs, and microbiota transplant resulted in aggravated clinical and pathological severity, reflecting increased alpha-synuclein aggregations and neuroinflammation in both the ENS and CNS of transgenic *SNCA* mouse models [113, 169].

### PRKN, PINK1, DJ-1

*PRKN*, *PINK1,* and *DJ-1* are the main disease-causative genes for autosomal recessive parkinsonism [168]. They function in maintaining mitochondrial homeostasis. Parkin, which is encoded by *PRKN*, is a E3 ubiquitin ligase that can be activated by PTEN-induced kinase 1 (PINK1) in response to damaged mitochondria to ubiquitinate mitochondrial surface proteins, leading to mitophagy [197]. DJ-1 is a sensor of oxidative stress that works in parallel to Parkin and PINK1 to protect against mitochondrial fragmentation [197]. Although mutations in *PRKN* and *PINK1* cause high penetrance of PD in mutation carriers, transgenic animal models with mutant *PRKN* and *PINK1* present minimal motor dysfunction, Lewy body pathology, and neuronal loss [198]. Intriguingly, a recent study demonstrated that intestinal bacterial infection triggers parkinsonian pathological changes and clinical motor symptoms in *PINK1* knock out (*PINK1*^−/−^) mice through neuroinflammation related to mitochondrial antigen presentation in immune cells, which were usually inhibited by *PRKN* and *PINK1* expression [173, 199]. Furthermore, synaptic dysfunction was observed in response to systemic rotenone intoxication in *PINK1*^±^ mice, possibly resulting from increased oxidative stress [172]. Thus, genetic susceptibility leading to mitochondrial dysfunction alters cellular responses to stress from environmental pathogens and toxins, which further enhances neuroinflammation and neurodegeneration. In addition, alteration of the gut microbiota and microbial metabolites in PD patients with *PRKN* or *LRRK2* mutation and in DJ-1-deficienct rodent models suggests an interplay between host genetics and microbiota in the pathophysiology of PD [164, 200, 201].

## Conclusions

Two decades after the dual-hit hypothesis proposed by Braak and colleagues, rapidly emerging evidence has validated the essential role of the gut microenvironment in the pathophysiology of PD as either one of the triggers or an aggravator of disease pathophysiology in those with “body-first” subgroup of PD patients. With advances in technology, elucidating the complex interaction between genetic risk factors with disease-prone gut microenvironmental changes unveils the heterogeneous features and pathophysiology of PD. Interventions targeting gut microbial changes could potentially modify the course of PD through gut-brain axis. A recent in vitro study provided evidence of two gut bacterial strains, *Parabacteroides distasonis* (MRX0005) and *Megasphaera massiliensis* (MRX0029) having anti-inflammatory and antioxidant capacity to mitigate neurodegeneration. The pleiotropic nature of these two potentially live biotherapeutic strains promoted a step into a Phase I clinical trial in PD patients in the coming future (https://parkinsonsnewstoday.com/news/phase-1-trial-will-evaluate-first-live-biotherapeutics-parkinsons-patients/). Because of the accumulating evidence from human and animal studies showing alterations in the gut microbiota in PD and the importance of the gut–brain axis in the PD process, future gut-oriented strategies to harmonize the gut microenvironment may have the potential to stop or halt the disease process at the very beginning of the prodromal gut stage of PD.

## Data Availability

Any data not published within the article are available from the corresponding author on reasonable request.

## Abbreviations

PD: Parkinson’s disease
AA: Acetic acid
BA: Butyric acid
CA: Cholic acid
CDCA: Chenodeoxycholic acid
CSF: Cerebrospinal fluid
DCA: Deoxycholic acid
GCA: Glycocholic acid
GCDCA: Glycochenodeoxycholic acid
GDCA: Glycodeoxycholic acid
GDCS: Glycodeoxycholic acid 3-sulfate
GLCAS: Glycolithocholic acid 3-sulfate
GUDCA: Glycoursodeoxycholic acid
HA: Hippuric acid
3-HK: 3-Hydroxykynurenine
H-Y stage: Hoehn and Yahr stage
IAA: Indole-3-acetic acid
ILA: Indolelactic acid
IS: Indoxyl sulfate
LCA: Lithocholic acid
PA: Propionic acid
TCA: Taurocholic acid
TCAS: Taurocholic acid 3-sulfate
TCDCA: Taurochenodeoxycholic acid
TDCA: Taurodeoxycholic acid
TMA: Trimethylamine
TMAO: Trimethylamine *N*-oxide
Trp: Tryptophan
TUDCA: Tauroursodeoxycholic acid
UDCA: Ursodeoxycholic acid
VA: Valeric acid
